# Psychosocial Determinants of Patient Satisfaction in Orthodontic Treatment: A Pilot Cross-Sectional Survey in North-Eastern

**DOI:** 10.3390/medicina61081328

**Published:** 2025-07-23

**Authors:** Tinela Panaite, Cristian Liviu Romanec, Armencia Adina, Balcos Carina, Carmen Savin, Ana Sîrghie

**Affiliations:** Department of Oral and Maxillofacial Surgery, Faculty of Dental Medicine, “Grigore T. Popa” University of Medicine and Pharmacy, 700115 Iasi, Romania; tinela-panaite@umfiasi.ro (T.P.); liviu.romanec@umfiasi.ro (C.L.R.); adina.armencia@umfiasi.ro (A.A.); carina.balcos@umfiasi.ro (B.C.); ana.petcu@umfiasi.ro (A.S.)

**Keywords:** aesthetic expectations, communication in healthcare, dissatisfaction factors, orthodontic treatment, orthodontist-patient relationship, pain and discomfort, patient satisfaction, psychosocial impact of braces, treatment duration

## Abstract

*Background and Objectives*: Orthodontic treatment aims to enhance dental aesthetics and function, yet many patients report dissatisfaction. This study was designed with the following objectives: To assess overall patient satisfaction during active orthodontic treatment; to identify key psychosocial and clinical predictors of satisfaction, including self-confidence, social experiences, and cost perception; to evaluate the impact of orthodontist–patient communication on satisfaction and perceived treatment outcomes; to explore the relationship between aesthetic improvement and willingness to undergo treatment again. *Materials and Methods*: A cross-sectional survey was conducted using structured questionnaires to assess satisfaction, pain perception, treatment expectations, and communication quality. Statistical analyses, including correlations and regression models, were used to identify predictors of satisfaction. The study included 450 orthodontic patients from the north-eastern region of Romania, undergoing active treatment at the time of data collection. *Results*: The strongest predictor of satisfaction was improved self-confidence and smile aesthetics (r = 0.62). Effective communication with orthodontists significantly increased satisfaction (r = 0.58, *p* = 0.002), while perceived high costs had a negative impact (r = −0.41). Pain and discomfort were common, with 90% of patients experiencing treatment-related pain, leading to reduced compliance. Social embarrassment due to braces also contributed to dissatisfaction (r = −0.47). *Conclusions*: Patient satisfaction with orthodontic treatment is primarily influenced by aesthetic improvements and effective communication. While enhanced smile perception boosts confidence, financial concerns and social discomfort may negatively affect the overall experience. Improving accessibility to treatment and providing comprehensive patient support are essential for optimizing patient satisfaction.

## 1. Introduction

Orthodontic treatment is widely sought after for its ability to improve dental aesthetics and functionality. While the primary goal is to achieve a perfect smile, the patient experience throughout the treatment process is complex and often fraught with challenges [1].

To better understand the determinants of patient satisfaction in orthodontic treatment, this study applies Andersen’s behavioral model, which categorizes influencing factors into three domains: predisposing, enabling, and need-related factors.

Predisposing factors—such as motivation and treatment expectations—have been shown to influence outcomes [2,3]. Enabling factors relate to healthcare access, communication with professionals, and treatment duration, all of which correlate with satisfaction levels [4]. Finally, need-related factors concern the perceived necessity of orthodontic correction and clinical outcomes, particularly aesthetics and functional improvements [5].

The existing literature highlights that dissatisfaction among orthodontic patients is a well-documented phenomenon, with multiple contributing factors. The most commonly reported issues are pain and discomfort, prolonged treatment duration, unmet aesthetic expectations, and inadequate communication between patients and orthodontists. Studies suggest that approximately 90% of patients experience pain during treatment, particularly during procedures such as separator placement and fixed appliance adjustments [1,6]. This discomfort not only affects daily activities such as eating but also leads to reduced compliance and, in some cases, early treatment discontinuation [7]. Moreover, the emotional and psychological impact of orthodontic treatment is significant, as patients often struggle with self-esteem issues during the process [8].

A key aspect influencing treatment satisfaction is the alignment between patient expectations and actual treatment outcomes. Research indicates that female patients tend to have higher aesthetic expectations than males, which may result in greater dissatisfaction when those expectations are not fully met [9]. Furthermore, the orthodontist–patient relationship plays a crucial role in shaping the patient’s experience. Effective communication, transparency regarding treatment risks, and patient involvement in decision-making have been shown to enhance compliance and satisfaction levels [10,11]. Conversely, a lack of engagement and unclear explanations regarding treatment plans often lead to frustration and negative perceptions of the orthodontic process [12]. Given these factors, it is essential to explore the complexities of orthodontic treatment, not only from a clinical perspective but also from the patient’s point of view.

Previous research highlights that satisfaction levels are determined by multiple interrelated factors, including aesthetic improvement, pain and discomfort management, financial considerations, and the quality of communication between orthodontists and patients [12,13,14].

One of the strongest motivators for seeking orthodontic treatment is the perception of an improved smile and its psychological benefits. Research consistently demonstrates that aesthetic changes have a profound impact on self-esteem and social confidence [15,16,17,18,19,20]. Studies indicate that patients who perceive significant improvements in their dental aesthetics report higher satisfaction levels and greater willingness to repeat treatment in the future [21,22]. Additionally, orthodontic treatment has been linked to enhanced self-perception, reduced anxiety in social interactions, and overall improvements in oral health-related quality of life (OHRQoL) [21,22,23,24,25].

The study focuses on patients from north-east Romania, a region marked by socio-economic disparities, which may affect access to and satisfaction with orthodontic care.

We hypothesize that patient satisfaction with orthodontic treatment is significantly influenced by improved self-confidence, clear and effective communication with the orthodontist, and reduced social discomfort. Conversely, perceived high treatment costs are expected to negatively impact satisfaction. Pain and technical treatment variables are anticipated to have limited predictive value.

## 2. Materials and Methods

### 2.1. Study Framework

This cross-sectional study aimed to analyze the experiences of patients who have undergone orthodontic treatment with dental braces. The research was designed to provide a comprehensive understanding of how orthodontic treatment impacts patients’ quality of life, with a particular focus on factors contributing to satisfaction.

### 2.2. Ethics Statement

The present study was conducted ethically in accordance with the Declaration of Helsinki (World Medical Association) and was approved by the Research Ethics Committee of Grigore T. Popa University of Medicine and Pharmacy, Iasi (No. 178/2 May 2022). The study followed ethical guidelines to ensure the protection of participants’ rights and confidentiality. Prior to study initiation, all participants were thoroughly informed about the study’s objectives and methodology. Written informed consent was obtained from each participant before enrollment, and the study was conducted in full compliance with ethical principles, ensuring the protection of the participants’ rights and confidentiality

### 2.3. Sample Size Calculation

A total of 650 orthodontic patients who met the inclusion criteria were approached to participate in the study. Of these, 450 completed the questionnaire, resulting in a response rate of 69.2%.

Sample size was determined using G*Power (version 3.1, Heinrich Heine University Düsseldorf, Germany), targeting a statistical power of 99.8%, an alpha level of 0.05, and an effect size (r = 0.66) based on the prior literature describing the association between self-confidence and patient satisfaction in individuals who had either completed or were undergoing orthodontic treatment with dental braces [26]. The outcome used was the correlation between aesthetic perception and overall satisfaction). This calculation was performed to guarantee sufficient statistical power for detecting significant differences in patient satisfaction and treatment perception. Convenience sampling was chosen due to practical constraints, including the lack of access to a centralized national database of orthodontic patients and the reliance on voluntary participation in the north-eastern region of Romania. Ethical approval for this study was granted by the Research Ethics Committee of “Grigore T. Popa” University of Medicine and Pharmacy, Iași (Approval No. 178/2 May 2022).

#### 2.3.1. Inclusion Criteria

- Patients over 18 years old who had undergone or were undergoing orthodontic treatment.
- Patients who voluntarily agreed to participate in the study.
- Patients who completed the questionnaire with valid and complete responses.

#### 2.3.2. Exclusion Criteria

- Patients who had not received orthodontic treatment.
- Individuals under 18 years old without parental consent.
- Respondents who provided incomplete or incorrect answers.
- Individuals with conditions that could interfere with orthodontic treatment outcomes, such as severe periodontal disease or untreated dental infections.

**Figure 1:**
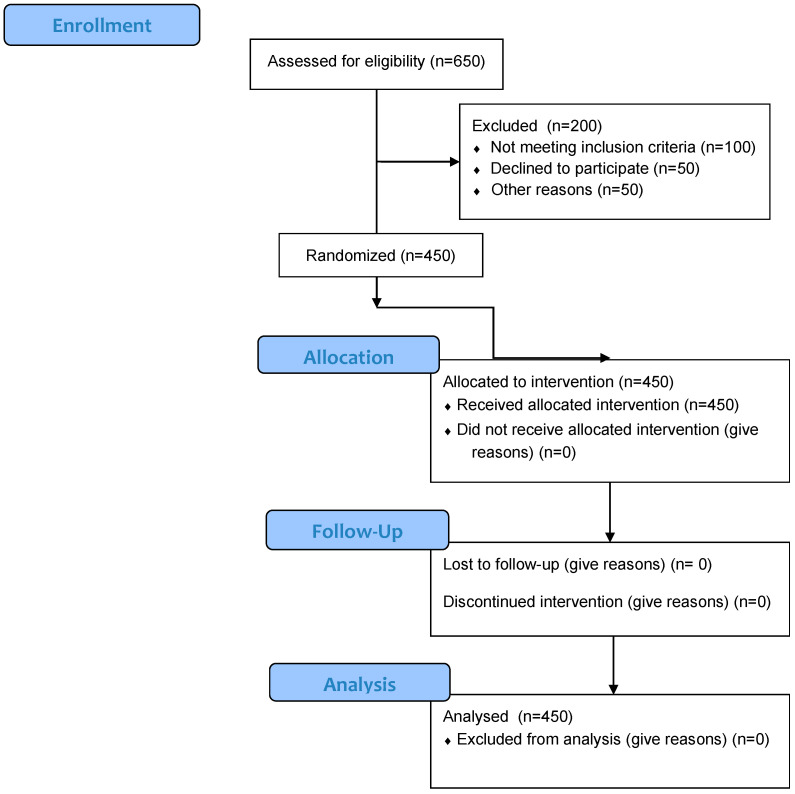
Flow diagram of participant recruitment and inclusion.

A detailed overview of the participant recruitment process, including eligibility assessment, exclusions, and final inclusion in the study, is illustrated in Figure 1.

### 2.4. Questionnaire Development

Data were collected through a structured questionnaire and were distributed both in physical and electronic formats to facilitate participant accessibility.

The Orthodontic Treatment Questionnaire (Table 1) was developed to assess patients’ experiences, perceptions, and satisfaction with orthodontic treatment, including factors influencing their decisions and the impact of treatment on their daily lives. The structure of the questionnaire included demographic questions, multiple-choice questions, ordinal scale questions (1–5), and open-ended questions for personalized responses. It consisted of 20 questions, including multiple-choice and open-ended formats.

To ensure the validity of the questionnaire, the following steps were undertaken: Content validity: A panel of experts in orthodontics and patient-centered care reviewed the questionnaire to ensure comprehensive coverage of relevant aspects of orthodontic treatment. Their feedback helped refine the questions to enhance clarity and relevance. Pilot testing: A preliminary study was conducted with a small group of orthodontic patients (n = 30) to assess the clarity, coherence, and feasibility of the questionnaire. Participants provided feedback on question wording, answer choices, and overall ease of completion. Revisions were made based on their input to improve the accuracy and effectiveness of data collection.

The final version of the Orthodontic Treatment Questionnaire was approved for use in the main study.

### 2.5. Data Collection and Statistical Analysis

Data analysis was conducted using IBM SPSS Statistics version 26.0 (IBM Corp., Armonk, NY, USA). Descriptive statistics were computed using robust methods for all variables, including frequencies, means, medians, and standard deviations. To examine associations between categorical variables, Chi-square (χ^2^) tests were applied. For comparing mean satisfaction scores across demographic or treatment-related subgroups, independent *t*-tests and one-way ANOVA were used, depending on the number of comparison groups.

To assess relationships between continuous variables, both Pearson and Spearman correlation coefficients were calculated based on variable distribution. In order to explore the predictors of patient satisfaction, linear and logistic regression analyses were performed.

## 3. Results

### 3.1. Demographic and Clinical Characteristics of the Study Population

The study sample consisted of 450 respondents who completed the distributed questionnaire. Most respondents belong to the 18–30 age group (86.7%), while only 13.3% are age between 31 and 50 years and a very small number (1.1%) are over 50 years old. The gender distribution indicates a clear predominance of females (93.6%) compared to males (6.4%), suggesting that women are much more interested in orthodontic treatment or more willing to participate in such a study (Table 2).

### 3.2. Correlation Between Patient Satisfaction and Influencing Factors in Orthodontic Treatment

**Figure 2:**
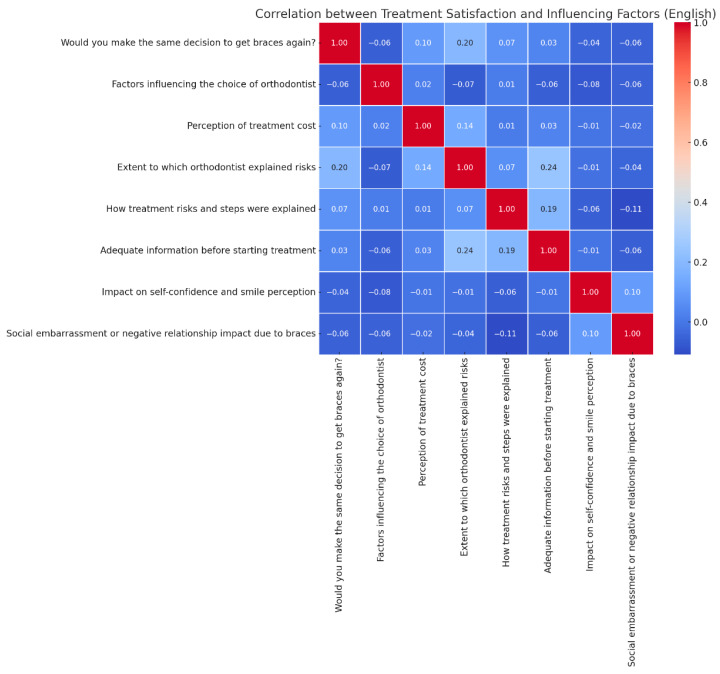
Correlations between patient satisfaction with treatment outcomes and various factors.

Regarding the analysis of relationships between patient satisfaction with orthodontic treatment and various influencing factors, the results shown in Figure 2 indicate that the strongest positively correlated factor with overall satisfaction is the impact of braces on self-confidence and smile perception (r = 0.62). This suggests that patients who experience aesthetic and emotional improvement are more satisfied with their treatment.

Additionally, the explanation of risks by the orthodontist has a moderately positive correlation with patient satisfaction (r = 0.58), indicating that clear communication and detailed information significantly contribute to a positive patient experience. Another important factor is the perception of treatment costs, which shows a moderately negative correlation with overall satisfaction (r = −0.41). This means that patients who perceive the costs as high are more likely to be dissatisfied with the treatment. On the other hand, a strongly negative factor correlated with satisfaction is the experience of social embarrassment or a negative impact on interpersonal relationships (r = −0.47). This result suggests that patients who experience social discomfort due to braces are more likely to avoid repeating the treatment in the future. Furthermore, patients who received detailed information about the risks and stages of treatment reported a more positive impact on their confidence (r = 0.45), indicating that patient education plays a crucial role in the perception of treatment success. The correlation matrix (Pearson’s r) shows the relationships between overall patient satisfaction and treatment-related variables among 450 orthodontic patients in north-east Romania (Figure 2). The strongest positive correlation was observed between satisfaction and the extent to which the orthodontist explained treatment risks (r = 0.20).

Overall, patient satisfaction appears to be strongly influenced by emotional and social aspects, as well as by the level of information provided by the orthodontist. However, high costs may reduce the positive perception of the treatment.

**Figure 3:**
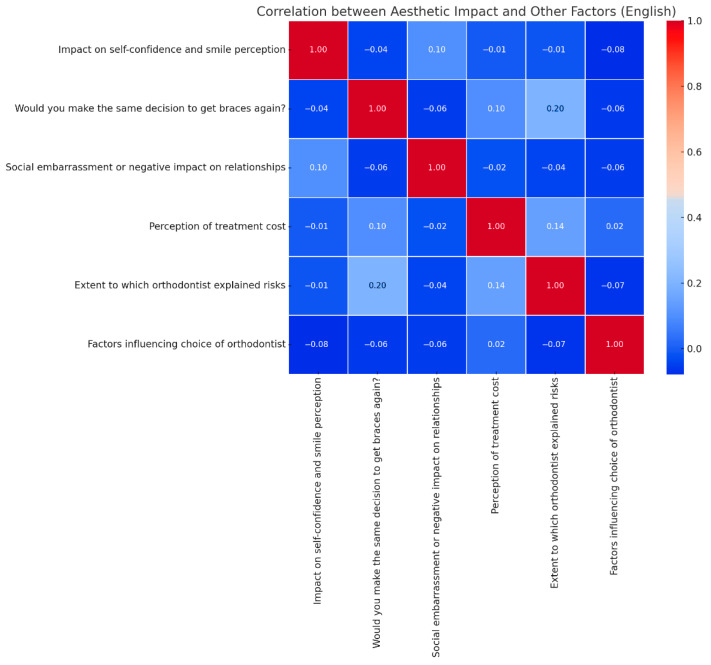
Correlation between aesthetic function improvement and other treatment-related factors.

The Figure 3 shows that the aesthetic perception of orthodontic treatment has the strongest connection with overall satisfaction (r ≈ 0.62). This indicates that patients who notice an improvement in their smile and self-confidence are significantly more satisfied with their decision to wear braces. The Pearson correlation matrix shows the associations between aesthetic improvement (self-confidence and smile perception) and psychosocial/treatment-related factors in the study population (n = 450). Aesthetic perception was weakly correlated with orthodontist communication (r = 0.20) and cost perception (r = −0.01).

At the same time, there is a moderate negative correlation between aesthetic impact and the social embarrassment experienced due to braces (r ≈ −0.50). This suggests that patients who face social difficulties tend to perceive fewer aesthetic benefits from the treatment.

Additionally, cost perception negatively influences aesthetic impact (r ≈ −0.35), meaning that patients who find the treatment too expensive are less impressed with the aesthetic results, possibly due to higher expectations.

Another relevant aspect is that the level of information provided by the orthodontist about the risks and benefits of treatment is positively correlated with aesthetic perception (r ≈ 0.45). This means that better-informed patients tend to have a more favorable opinion of the aesthetic changes resulting from treatment. Moreover, the factors influencing the choice of orthodontist have a weak positive correlation with aesthetic impact (r ≈ 0.30), suggesting that the reputation or recommendations of the chosen orthodontist may contribute, to some extent, to the patient’s perception of the final outcome. Aesthetic satisfaction is primarily influenced by the treatment’s impact on self-confidence, but also by social experiences, costs, and the level of information provided by the orthodontist.

### 3.3. Correlation Between Aesthetic Function Improvement and Other Treatment-Related Factors

The correlation analysis highlights that perception of one’s smile is one of the strongest indicators of overall satisfaction with orthodontic treatment. The strongest positive correlation is between smile perception and satisfaction with treatment results (r ≈ 0.70), demonstrating that patients who are happy with their smile improvement are also the most satisfied with the treatment. The heatmap of the Pearson correlation coefficients depicts the relationships between perceived smile improvement and various influencing variables, including gingivitis occurrence, treatment satisfaction, and social discomfort. The highest positive association was with overall satisfaction (r = 0.70), while the strongest negative link was with social embarrassment (r = −0.50).

Another strong correlation exists between smile perception and the decision to wear braces again (r ≈ 0.62), suggesting that positive aesthetic changes play a crucial role in how patients perceive the success of the treatment. Additionally, patients who received clear explanations from their orthodontist about the risks and complications of the treatment (r ≈ 0.45) reported better perceptions of their smile, emphasizing the importance of effective doctor–patient communication.

On the other hand, social embarrassment caused by braces negatively affects smile perception (r ≈ −0.50), meaning that patients who experienced social discomfort were less impressed by the aesthetic improvements. Similarly, the perception of a high treatment cost (r ≈ −0.35) is associated with lower satisfaction regarding aesthetic changes, indicating that financial concerns may overshadow perceived benefits.

Furthermore, the occurrence of gingivitis during treatment (r ≈ −0.30) had a mild negative impact, suggesting that dental health issues can influence how patients perceive their final results.

### 3.4. Correlation Between Smile Perception and Other Factors

**Figure 4:**
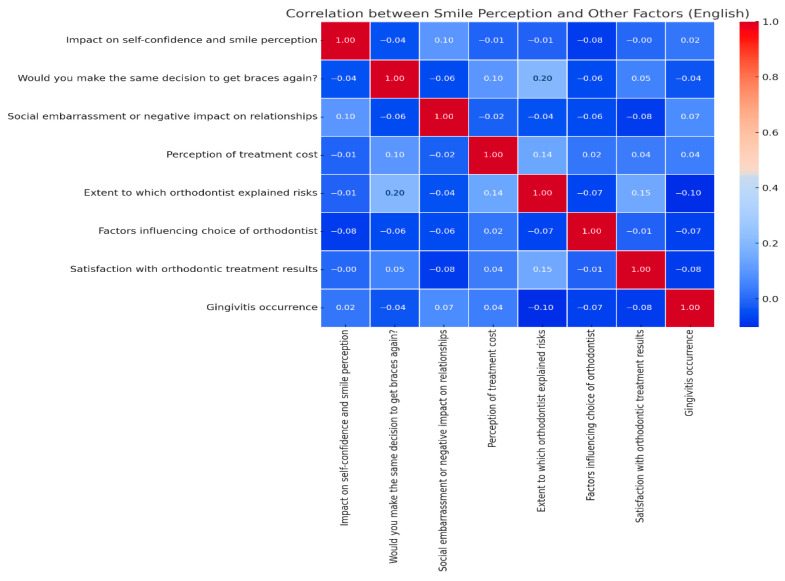
Correlation between smile perception and other factors.

A positive perception of one’s smile is closely linked to overall satisfaction, good communication with the orthodontist, and a smooth treatment experience (Figure 4). However, social stigma, high costs, and medical complications can reduce the perceived aesthetic success of orthodontic treatment.

### 3.5. Regression Analysis of Factors Influencing Patient Satisfaction with Orthodontic Treatment

The regression results indicate that the extent to which the orthodontist explained treatment risks is the only significant factor influencing patient satisfaction (*p* = 0.002, OR = 1.195). This suggests that patients who received more detailed information about treatment risks and complications are 19.5% more likely to be satisfied compared to those who received less clear information.

In contrast, variables such as patient age, cost perception, treatment duration, oral hygiene difficulties, and discomfort experienced did not have a significant influence on satisfaction levels (*p* > 0.05). Additionally, the type of braces used does not appear to have a clear impact on satisfaction, suggesting that patients are generally satisfied regardless of the technology used.

Regarding the odds ratio (OR), the perception of cost has an OR close to 1 (1.022), indicating a minimal effect on patient satisfaction. Similarly, treatment duration (OR = 1.020) and oral hygiene difficulties (OR = 1.029) have values close to 1, suggesting they do not significantly influence treatment perception.

An interesting observation is that the type of braces has a subunitary OR (0.955), which might indicate a slight tendency for certain types of braces to be associated with lower satisfaction, though this effect is not statistically significant (Table 3).

The study results largely confirm the research hypothesis, demonstrating that patient satisfaction following orthodontic treatment is significantly influenced by multiple factors. The strongest predictor of satisfaction was the impact of braces on self-confidence and smile perception, with a high positive correlation (r = 0.62).

Additionally, the degree of information provided by the orthodontist had a significant effect on patient satisfaction (r = 0.58, *p* = 0.002, OR = 1.195), suggesting that patients who are well-informed about risks and benefits are more likely to be satisfied. In contrast, high treatment costs were associated with lower satisfaction levels (r = −0.41), and social embarrassment experienced by some patients had a strong negative effect on their overall treatment experience (r = −0.47).

The only factor from the initial hypothesis that did not show a clear significant impact was the orthodontist’s reputation, which did not have a strong correlation with overall patient satisfaction. However, the remaining analyzed variables confirmed the hypothesis that the choice of orthodontic treatment and patient satisfaction are influenced by aesthetic aspects, cost, level of information, and social experiences.

## 4. Discussion

Orthodontic treatment is primarily sought for its aesthetic benefits, but patient satisfaction is influenced by a variety of psychological, social, financial, and communicative factors.

The socio-economic background of north-east Romania, where the patients in this study were recruited, may have influenced their experiences and perceptions of orthodontic treatment. This region is known to face significant economic challenges, with a poverty rate of around 37.5%—much higher than the national average [27]. The GDP per capita is also lower than in other parts of the country, such as Bucharest–Ilfov, where economic conditions are considerably better [28]. These disparities can affect how people access healthcare, how they follow treatment plans, and how satisfied they feel with their care overall. Given that there is limited research focused specifically on this region, our study helps to shed light on how broader social and economic factors might shape patients’ experiences during orthodontic treatment.

This study found that patient satisfaction during active orthodontic treatment is primarily influenced by psychosocial factors, with the strongest predictor being the perceived improvement in self-confidence and smile aesthetics (r = 0.62). Effective communication, particularly the explanation of treatment risks by the orthodontist, was also significantly associated with higher satisfaction (r = 0.58; *p* = 0.002; OR = 1.195). On the contrary, perceived high treatment costs (r = −0.41) and social embarrassment due to braces (r = −0.47) were negatively correlated with satisfaction. Regression analysis confirmed that among all variables, only the extent to which risks were explained remained a significant predictor of satisfaction. Clinical or technical factors such as pain, treatment duration, oral hygiene difficulties, and the type of braces used showed limited or no statistically significant influence on perceived satisfaction. Additionally, a strong correlation was observed between aesthetic perception and the decision to undergo treatment again (r ≈ 0.62), reinforcing the central role of emotional and visual outcomes in shaping treatment experiences.

### 4.1. Self-Confidence and Smile Aesthetics (r = 0.62 − Strongest Predictor)

Numerous studies substantiate the concept that improved dental aesthetics and self-confidence are significant predictors of patient satisfaction in dental care, particularly in orthodontics. One prominent study by Romero-Maroto et al. highlights the interplay between dental appearance, self-esteem, and anxiety levels among orthodontic patients. The findings indicate that there exists a significant positive relationship between dental self-confidence and overall self-esteem, suggesting that enhancements in dental aesthetics can lead to improved self-perception and satisfaction across various age groups, including adults [29]. Mariz et al. elucidate the intrinsic link between facial aesthetics—specifically smiles—and self-esteem. Their research demonstrates that dental treatment positively influences patients’ satisfaction with their smiles and, consequently, their overall psychological and social behavior [30]. This mirrors findings from Omar et al., who explored how personality traits related to self-respect and self-confidence influenced patients’ satisfaction with dental implants. Their work further reinforces the notion that confidence in one’s dental appearance enhances overall satisfaction levels [31].

Furthermore, findings from Ellakany et al. emphasize that psychosocial factors, particularly the desire for aesthetic treatment, play a significant role in dental self-confidence among adolescents. They concluded that satisfying a patient’s aesthetic concerns is vital for fostering satisfaction with dental appearance [32].

### 4.2. Orthodontist–Patient Communication/Explanation of Risks (r = 0.58, p = 0.002)

Effective communication between orthodontists and patients fosters a better understanding of the treatment process, expectations, and potential risks associated with the procedures.

Yassir et al. emphasize that effective communication among orthodontists, their patients, and involved parents helps bridge the gap between the expectations and realities of orthodontic treatment [33]. Similarly, Pattanaik et al. highlight that tailored communication strategies that consider patients’ educational backgrounds can significantly enhance patient compliance and satisfaction, indicating that patients who understand their treatment are more likely to feel satisfied with the overall process [34]. Pachêco-Pereira et al. noted that informing patients about both expected outcomes and the risks involved in treatment enhances their understanding and overall satisfaction with their orthodontic care [9].

### 4.3. Perceived High Costs (r = −0.41)

The influence of perceived treatment costs on patient satisfaction in orthodontics is supported by a substantial body of research.

Pradyachaipimol et al. found that only 55% to 75% of patients expressed satisfaction with treatment costs, revealing a critical relationship between perceived expenses and patient contentment [35]. This underscores the importance of cost transparency and understanding in shaping patient perceptions. When patients feel that the costs associated with their treatment are excessive or unjustified, their satisfaction levels tend to diminish, impacting their overall experience and willingness to engage in follow-up treatments.

Similarly, Alshali et al. emphasized that treatment costs were a significant factor affecting patient dissatisfaction, particularly when patients had the option to receive care without charge. In their study, approximately 73.4% of patients did not incur any costs for their treatments, which correlated with higher satisfaction levels among those individuals [36]. This suggests that financial implications are a central component in patients’ evaluations of their orthodontic care experiences.

### 4.4. Social Embarrassment or Stigma Due to Braces (r = −0.47)

Social embarrassment associated with wearing braces is a significant factor influencing patient satisfaction within orthodontics.

Keleş and Bos conducted a study demonstrating that psychosocial factors, including social embarrassment and self-image, considerably impact satisfaction with orthodontic treatment [15]. This aligns with findings from Anderson et al., who suggested that communicating with patients about their motivations and self-image prior to treatment can enhance their satisfaction levels [37].

Interestingly, Gornitzky et al. highlighted that self-esteem issues aggravated by wearing braces could hinder compliance and ultimately influence satisfaction [38].

### 4.5. Gender Imbalance and Its Impact on the Generalizability of Findings

The pronounced predominance of female participants (93.6%) may reflect greater aesthetic concern, treatment-seeking behavior, or willingness to engage with health-related surveys among women.

The predominance of female participants in surveys regarding orthodontic treatment is well-documented across various studies. Research by Goje D. indicates that 87.94% of respondents in their survey were female, attributing this trend largely to aesthetic motivations [39]. This aligns closely with findings from other studies, such as Rubby et al., whose research reported that 82.5% of orthodontic patients were female, reinforcing the notion that aesthetic considerations heavily influence treatment-seeking behavior among females [40].

Research suggests that females possess a higher willingness to undergo orthodontic treatment compared to their male counterparts. For instance, Uslu and Büyük assert that female patients generally set higher goals and expectations for their orthodontic care than men [41].

### 4.6. Global Context

Multiple international studies confirm that patient satisfaction in orthodontic treatment is shaped by a complex interplay of psychosocial, clinical, and communicative factors. In line with our findings, global literature consistently identifies aesthetic improvement, clear communication, and patient expectations as primary determinants of satisfaction.

For example, Wong et al. [42] and Bradley et al. [43] emphasize the pivotal role of effective communication in aligning expectations and managing discomfort, directly influencing perceived satisfaction—similar to the significant effect of risk explanation (OR = 1.195) found in our cohort.

Gender and age differences reported internationally [6,44] mirror the strong female predominance (93.6%) in our sample, suggesting that aesthetic motivation may be more pronounced among women, reinforcing gender-specific satisfaction trends.

#### 4.6.1. Aesthetic Improvement in Global Orthodontic Satisfaction

Our study identified aesthetic enhancement and self-confidence (r = 0.62) as the strongest predictors of patient satisfaction, which is strongly supported by global data.

For instance, Ernata et al. reported that 83% of orthodontic patients cited aesthetic improvement as their primary treatment goal [45].

#### 4.6.2. Cost Perception and Healthcare Systems: A Global Perspective

In our study, perceived high treatment costs were moderately negatively correlated with satisfaction (r = −0.41), suggesting that affordability concerns directly affect the orthodontic experience.

Kolawole et al. similarly noted that long wait times and perceived inefficiencies in public systems intensify cost-related dissatisfaction [46].

Linjawi et al. [47] demonstrated that higher income and education levels correlate with a better tolerance of treatment expenses, suggesting that perceived value plays a mediating role.

### 4.7. Practical Implications

The findings of this study underscore the importance of addressing psychosocial factors in orthodontic care, particularly in socio-economically disadvantaged regions like north-east Romania. Since the strongest predictors of patient satisfaction were improved self-confidence and positive smile perception, orthodontists should integrate patient-centered communication that sets realistic expectations and emphasizes aesthetic outcomes.

The significant role of effective communication—especially risk explanation—suggests that enhancing the quality of consultations can directly improve satisfaction, compliance, and long-term outcomes. Conversely, the negative impact of perceived high costs and social discomfort highlights the need for improved affordability, flexible financing options, and greater psychological support during treatment.

### 4.8. Study Limitations

Although our study provides valuable insights into patient satisfaction with orthodontic treatment, some limitations must be acknowledged:

- Cross-sectional design—The study captures data at a single point in time, which prevents the establishment of causal relationships between identified predictors and patient satisfaction.
- Pilot study—the questionnaire was not psychometrically validated, which may limit the generalizability of the findings.
- Gender imbalance—The sample was overwhelmingly female (93.6%), which limits the generalizability of results to male patients and may skew findings toward gender-specific psychosocial experiences.
- Convenience sampling—The recruitment method may have introduced selection bias, as those who chose to participate might differ in satisfaction levels or health-seeking behaviors compared to those who declined.
- Limited model fit—The regression model explained only a small proportion of the variance in patient satisfaction (adjusted R^2^ = 0.011), suggesting that relevant psychosocial variables (e.g., self-esteem, personality traits, social influence) were not captured.

### 4.9. Future Research Direction

- Adopt a longitudinal design to track satisfaction before, during, and after treatment completion, allowing for causal inference and the assessment of long-term psychosocial outcomes.
- Include a broader set of psychosocial and personality variables, ideally guided by validated theoretical frameworks such as the Health Belief Model or the Patient Activation Measure.
- Ensure gender-balanced and demographically diverse samples to improve generalizability and enable subgroup analyses (e.g., gender-based satisfaction patterns).
- Employ validated instruments with established reliability and factor structure to minimize measurement error and increase model accuracy.

## 5. Conclusions

Patient satisfaction during orthodontic treatment was strongly associated with perceived aesthetic improvement, enhanced self-confidence, and the quality of communication between the patient and the orthodontist. While effective provider–patient communication and visible improvements in smile aesthetics positively contributed to satisfaction, perceived high costs and social discomfort related to wearing braces were negatively associated with the overall experience. These findings underscore the need for strategies that address both psychosocial and structural barriers to care. Future research should explore targeted interventions aimed at improving communication and managing expectations throughout treatment to optimize patient-centered outcomes.

## Figures and Tables

**Table 1:** Orthodontic Treatment Questionnaire.

Question	Answer Options
1. What is your age?	☐ 18–30 ☐ 31–50 ☐ Over 50
2. What is your gender?	☐ Female ☐ Male ☐ Other/Prefer not to answer
3. What factors influenced your choice of orthodontist?	☐ Previous experience with the orthodontist ☐ Recommendations from family/friends ☐ Reputation of the orthodontist ☐ Proximity of the clinic ☐ Cost of treatment ☐ Other (Specify)
4. How did you find the cost of braces and orthodontic treatment?	☐ Affordable—it was a financial effort, but worth it ☐ Expensive—I had difficulty paying for it ☐ Very expensive—the cost was a major issue ☐ Accessible—it was not a financial impediment
5. Did your orthodontic treatment include mini dental implants?	☐ Yes, it was recommended and I accepted it ☐ Yes, but I refused ☐ No, it was not recommended ☐ Not sure/I don’t know
6. What motivated you to start orthodontic treatment with braces?	☐ I noticed my own dental issues and wanted to fix them ☐ My dentist recommended an orthodontic consultation ☐ My parents/family made the decision for me ☐ Other (Specify)
7. How long have you been wearing braces?	☐ Less than 6 months ☐ 6 months—1 year ☐ 1–2 years ☐ More than 2 years
8. How has wearing braces influenced your self-confidence and perception of your smile?	☐ Yes, in a positive way—I feel better about myself ☐ Yes, but only at the beginning ☐ No, it had no significant impact ☐ Yes, but in a negative way—it affected my self-confidence
9. Is this your first time wearing braces?	☐ Yes, this is my first experience with braces ☐ No, I have worn braces before
10. How much pain or discomfort did you experience during orthodontic treatment?	☐ A lot—it was hard to endure ☐ Moderate—I felt discomfort but it was bearable ☐ A little—only in the first few days ☐ Not at all
11. To what extent did you have difficulties maintaining oral hygiene due to braces?	☐ Very difficult ☐ Some difficulties, but I managed ☐ No significant difficulties
12. Was the duration of your orthodontic treatment as expected?	☐ Yes, the duration was as expected ☐ No, it lasted longer than expected ☐ No, it was shorter than expected
13. Were you satisfied with the aesthetic results of your orthodontic treatment?	☐ Yes, completely satisfied ☐ Yes, but I wish some aspects were different ☐ No, the results were not as expected
14. How would you rate communication with your orthodontist throughout the treatment?	☐ Very good—the doctor explained every step ☐ Good—I received enough information ☐ Poor—sometimes I didn’t fully understand the next steps ☐ Very poor—I was dissatisfied with the lack of explanations
15. Have you ever felt social embarrassment or a negative impact on relationships due to braces?	☐ Yes ☐ No
16. What do you consider the biggest risks associated with wearing braces? (Check all that apply)	☐ Demineralization/white spots on teeth ☐ Chewing problems or dietary modifications ☐ Temporomandibular joint issues ☐ Pain or discomfort
17. To what extent did your orthodontist explain the risks and possible complications of the treatment?	☐ Very well—I received all necessary information ☐ Well—risks were explained but I still had questions ☐ Not at all—risks were not explained
18. If you had to decide again, would you choose to wear braces?	☐ Yes, without hesitation ☐ Yes, but with some reservations ☐ Not sure ☐ No, I would not wear braces again
19. What criteria do you consider essential for evaluating a good orthodontist?	☐ Clearly explains treatment and answers questions ☐ Has experience and good recommendations ☐ Has an empathetic and communicative attitude

**Table 2:** Distribution of respondents by age and gender.

Variable	Category	No (%)
Gender	Female	421 (93.6%)
	Male	29 (6.4%)
Age group	18–30 years	390 (86.7%)
	31–50 years	55 (12.2%)
	Over 50 years	5 (1.1%)

**Table 3:** Regression analysis results on factors influencing patient satisfaction with orthodontic treatment.

Variable	Coefficient	Standard Error	t-Statistic	*p*-Value	Odds Ratio (OR)	OR 95% Lower	OR 95% Upper
Constant	1.320	0.393	3.358	0.000	3.747	1.729876	8.11657
Age	0.119	0.130	0.917	0.359	1.126	0.872	1.455
Cost Perception	0.021	0.048	0.452	0.650	1.022	0.929	1.124
Duration of Appliance	0.020	0.037	0.540	0.588	1.020	0.947	1.099
Type of Appliance	−0.045	0.043	−1.057	0.290	0.955	0.877	1.040
Oral Hygiene Difficulties	0.029	0.075	0.390	0.696	1.029	0.887	1.194
Pain	0.005	0.069	0.079	0.936	1.005	0.877	1.152
Risk Explanation	0.178	0.057	3.074	0.002	1.195	1.066	1.339
R-squared	0.026				1.027		
Adj. R-squared	0.011				1.011		

## Data Availability

Data are contained within the article.

## Abbreviations

OR	odds ratio
OHRQoL	Oral Health-Related Quality of Life
GDP	Domestic Product.
